# Anti-Wrinkle and Skin Moisture Efficacy of 7-MEGA^TM^: A Randomized, Double-Blind, Placebo Comparative Clinical Trial

**DOI:** 10.3390/nu16020212

**Published:** 2024-01-09

**Authors:** Hyun Kyung Sung, Tae Jeong Kim, Hyung Mook Kim, Sang Jun Youn, Yong Choi, Na Young Lee, Hyun Jeong Oh, Hyuck Se Kwon, Seon Mi Shin

**Affiliations:** 1 Global Cosmeceutical Center, Semyung University, Cheongju 28161, Republic of Korea; 2 Department of Pediatrics, College of Korean Medicine, Semyung University, Jecheon 27136, Republic of Korea; 3 Department of Education, Graduate School, Dongguk University, Seoul 04620, Republic of Korea; 4 Department of Pediatrics, Semyung University Chungju Korean Medicine Hospital, Chungju 27429, Republic of Korea; 5 RnBS Corporation, Seoul 06032, Republic of Korea; 6 R&D Team, Food & Supplement Health Claims, Vitech, Wanju 55365, Republic of Korea; 7 Department of Internal Medicine, College of Korean Medicine, Semyung University, Jecheon 27136, Republic of Korea

**Keywords:** omega-7, 7-MEGA^TM^, randomized controlled trial, skin nutritional supplements, skin aging

## Abstract

7-MEGA^TM^ is a food product made from purified Alaska pollack fish oil containing palmitoleic acid (16:1), commonly referred to as omega-7. We sought to quantitatively evaluate whether this substance inhibits skin aging. A total of 101 middle-aged females were randomly allocated to the intervention (*N* = 50) or placebo group (*N* = 51). Each participant was advised to take either 500 mg of 7-MEGA^TM^ or a placebo twice daily for 12 weeks. The primary outcomes were the degree of improvement in wrinkles and the degree of moisture filling after consumption for 12 weeks compared to baseline. The secondary outcomes were improvement in skin wrinkles; moisture changes at 4 and 8 weeks from baseline; changes in transdermal water loss, skin elasticity, the melanin index, the erythema index, and the Global Photo Damage Score. We found a significant improvement in skin wrinkles and elasticity at 12 weeks in the 7-MEGA^TM^-consuming group compared to that in the placebo group; skin moisture, elasticity, and the melanin index were also improved. No supplement-related adverse reactions were observed and 7-MEGA^TM^ was identified as safe. 7-MEGA^TM^ was effective for human skin function in terms of wrinkles, moisture, elasticity, and melanin production and may be useful as a skin nutritional supplement.

## 1. Introduction

7-MEGA^TM^ is a food manufactured from refined Alaskan pollock fish oil containing palmitoleic acid (PA; 16:1), which is an unsaturated fatty acid also called omega-7 because it has a double bond at the 7th carbon. It can be consumed through the diet or converted from components, such as fats and carbohydrates that are already present in the body [1]. Consumption of omega-7 improves mucosal hydration and regenerates the skin; in the case of metabolic syndrome, it increases high-density lipoprotein cholesterol levels and helps improve insulin sensitivity [2]. However, there have been few studies conducted on the relationship between skin aging, such as anti-wrinkle efficacy and 7-MEGA^TM^.

The skin occupies approximately 1.7 m^2^ in area of the human body [3] and performs various roles in addition to aesthetic functions. It consists of four layers: the stratum corneum, which is located on the outer skin and is the residue from which the epidermis sheds; the epidermis and dermis; and the subcutaneous tissue, which forms the base layer [4]. These four skin layers act as an environmental barrier that protects major organs, helps regulate temperature, and functions as an immunological factor axis [5].

The subcutaneous tissue layer undergoes multiple changes that contribute to the aging appearance of the face, including decreased adipocyte size, function, and differentiation and impairment of adipocyte redistribution during intrinsic aging [6]. In general, facial wrinkles become more pronounced as we age because they are the result of the facial muscles repeating the same actions. As a result, the faces of middle-aged people are characterized by deep wrinkles on the forehead, between the eyebrows, and around the eyes [7].

In addition to wrinkles, skin aging also results in the loss of skin moisture. The surface of the epidermis goes through several differentiations to produce the stratum corneum, which is a water retention area. Proliferation, differentiation, and exfoliation of one layer of keratinocytes occur constantly, with the epidermis below forming one layer. The stratum corneum is composed of a mixture of keratinocytes, fatty acids, and intercellular lipids of cholesterols [8]. The intracellular space of these keratinocytes is filled with a water-soluble degradation product of filaggrin, consisting of components such as amino acids and lactic acid. Thus, it absorbs water well, effectively binding water in a dry environment [9]. However, the moisture content of the stratum corneum decreases with age [10].

Abnormal pigmentation, represented by melanin and hemoglobin, is also evidence of skin aging. Melanin is located in the approximately top 50–100 µm of the epidermis [11], is differentiated from melanocytes, and protects the skin. In aging melanocytes, melanin synthesis, autophagy activity, and melanin pigmentation decrease; therefore, skin pigmentation tends to increase as aging accelerates [12]. Furthermore, aging skin lacks redness compared to young skin because the vascular activity of the dermis is low and thus appears pale [13].

In the field of dermatology, physical and chemical interventions and prevention are performed to prevent skin aging. Mechanically, thermal devices, such as laser, high-intensity focused ultrasound, and radiofrequency, are used to improve elasticity and wrinkles [14]. Topical agents, such as retinoids, vitamin C, and hyaluronic acid injections, are also effective in improving elasticity, synthesizing collagen, and hydrating skin tissue [15,16]. Other edible foods, such as collagen supplements, bird nests, and BB-1000, are known to affect skin aging and wrinkles [17,18,19].

The purpose of this trial was to provide scientific evidence for 7-MEGA^TM^ as a skin health functional material and confirm the improvement effects of 7-MEGA^TM^ on skin wrinkles, moisture, elasticity, and melanin and erythema production. We found that 7-MEGA^TM^ improved skin health through the skin wrinkle, skin moisturizing, and skin elasticity function improvement effects and the melanin index improvement effect.

## 2. Materials and Methods

### 2.1. Study Design

We evaluated the functionality and safety of 7-MEGA^TM^ for skin health in healthy adult females aged 40 to 59 years recruited at the Chungbuk Cosmetics Clinical Research Support Center (Cheongju, Chungcheongbuk-do, Republic of Korea).

A screening test was conducted on 109 human participants. Eight participants were excluded, and 101 were randomly assigned. Initially, 50 females were assigned to the 7-MEGA^TM^ group and 51 to the control group. However, during the trial, three participants dropped out of the intervention group, and 98 participants completed the human application test (47 in the 7-MEGA^TM^ group and 51 in the control group).

Participants were randomly allocated to orally consume 7-MEGA^TM^ (purified Alaskan pollock fish oil containing PA) or a placebo for 12 weeks, and tests were performed to evaluate the effects on skin wrinkles, elasticity, moisture, and melanin and erythema production. The first human application test subject was registered on 13 March 2023, and the last human application test subject was examined on 19 June 2023.

We submitted our protocol to the Institutional Review Board (IRB) of Jecheon Semyung University Korean Medicine Hospital and the protocol was approved before the start of the trial (IRB No.: SMJOH-2023-03). This clinical trial was registered with the Korean Clinical Research Information Service (PRE20231115-005). The selection and exclusion criteria for the participants are presented in Table 1.

### 2.2. Intervention

One capsule (1000 mg) of the test food contained 500 mg of refined Alaskan pollock fish oil (7-MEGA^TM^) containing PA; it was a soft capsule with a unique color and flavor and no unpleasant odor. The control food was manufactured in the same shape and color as the test food, making it difficult to distinguish between the two products. The control food did not contain PA-containing refined Alaskan pollock fish; its main ingredient was corn oil.

The participants were instructed to take one capsule orally twice a day, after breakfast and dinner, and to maintain their usual lifestyle habits. In addition, participants visited the hospital at weeks 4, 8, and 12 of the medication administration to check medication compliance and unused tablets were collected.

While participating in the trial, the use of medications with the risk of interfering with the trial results was prohibited. At every visit, the cosmetics and skin care devices that the participants used were investigated and recorded (only products that could improve wrinkles, moisturize, and affect elasticity were recorded in the case record). During the trial, participants were instructed not to use any functional cosmetics that could improve skin wrinkles (retinoids, alpha hydroxy acid, etc.), skin moisture, and skin elasticity, and were instructed not to change the cosmetics they were using if possible. If there were changes, all cosmetic product names and periods of use were recorded.

If any special conditions or situations occurred during the trial, the participant was removed from further participation in the study. The situations were as follows: a serious adverse reaction causing hospital admission, application of medication or medical treatment that could affect the skin condition, withdrawal of consent to participate in the clinical trial, violation of dosage or visit date, and loss of contact or traceability.

### 2.3. Block Randomization and Participant Blinding

In this study, block randomization was used to ensure a balanced allocation to prevent possible allocation bias and increase comparability between the groups. If a participant satisfied the inclusion/exclusion criteria in the screening test and was determined suitable for the human application test, block randomization was performed. During the random assignment, the 7-MEGA^TM^ and control groups were assigned in an equal ratio of 1:1. The randomization assignment was registered by assigning a random code to each participant using a web-based automatic response system (interactive web response system [IWRS]). After entering the participant number into the IWRS, the investigator assigned an Investigational Product (IP) number to the participant and delivered the product corresponding to the IP number of the system. The participants and research team were blinded to the packaging, except for individual IP numbers, so that each group could not be identified by the packaging. If the trial product was defective or damaged, a new IP number was assigned, corresponding to the initial group. Both the code and number were accessed only by a person who had no relationship with the trial and were sealed until the end of the trial, except when explicit verification was required.

### 2.4. Outcomes

To evaluate skin functionality, skin wrinkles were evaluated using the SV700 (Courage + Khazaka Electronic GmbH, Köln, Germany), skin moisture content was evaluated using the corneometer (Corneometer CM 825; Courage + Khazaka Electronic GmbH), transepidermal water loss was evaluated using the tewameter (Tewometer TM 300; Courage + Khazaka Electronic GmbH), skin elasticity was evaluated using the cutometer (Cutometer MPA 580; Courage + Khazaka Electronic GmbH), melanin and erythema production was evaluated using the mexameter (Mexometer MX 18; Courage + Khazaka Electronic GmbH), and skin wrinkles were also visually evaluated by experts.

The primary outcomes of the trial were changes or improvements in wrinkles and moisture content. Skin wrinkles were measured 2–3 cm away from the corners of both eyes using the SV700 and were divided into five parameters: R1, skin roughness, referred to the difference between the highest and lowest wrinkle roughness peaks; R2, the maximum roughness, referred to the largest R1 value among the roughness sections divided into five equal lengths; R3, the average roughness, was the arithmetic mean of the single roughness depths of each of the five subunits; R4, the smoothness depth, referred to the average depth of skin wrinkles; and R5, the arithmetic roughness average, referred to the arithmetic average of the peaks of the roughness cross-section of the wrinkles. For both outcomes, the values at week 12 were compared with those at the initial visit.

Skin moisture is evaluated using a corneometer CM 825, which measures the capacitance of the current transmitted through a probe in contact with the skin. The moisture and capacitance are proportional to each other; therefore, the higher the moisture content, the higher the measured value. Skin moisture was measured at the intersection of both eye corners and the nose tip (cheek) and 10 cm inside the left and right wrists (forearm).

The secondary outcomes were the change or improvement in skin wrinkles (R1–5); the amount of change in skin moisture measured at 4 and 8 weeks compared to the initial visit; and the amount of transepidermal water loss measured at 4, 8, and 12 weeks compared to baseline. As in the measurement of moisture using the corneometer, transepidermal water loss was measured at the intersection of both eye corners and the nose tip and 10 cm inside the left and right wrists (forearm). Evaluation of transepidermal water loss using a tewameter is a method for measuring skin barrier function. It is used as an indicator to measure the skin’s ability to recover after skin damage and the moisture content of the stratum corneum. As skin dryness increases, the measured value increases.

The amount of change in skin elasticity was measured using three parameters: R2, the overall elasticity value; R5, the actual elasticity value; and R7, the ratio of elasticity to the entire curve. The closer the value of each item was to 1, the higher the elasticity. Measurements were performed at the intersection of both eye corners and the nose tip using a cutometer MPA 580.

We also measured the amount of change in the melanin index, erythema index, and Global Photo Damage Score (GPDS). The melanin and erythema indexes are quantities of melanin and hemoglobin, which are major factors that determine skin color, and are quantified skin absorption rates in each wavelength region, measured through the mexameter MX 18 at the intersection of both eye corners and the nose tip. Visual evaluation was performed using the GPDS at Visit 1 (screening visit), Visit 2 (week 0), Visit 3 (week 4), Visit 4 (week 8), and Visit 5 (week 12). To evaluate the GPDS, we assessed the wrinkle state based on the judgement of two experts on a scale of 0 (none) to 7 (very severe). The experts independently evaluated wrinkles 2–3 cm away from the eye corners without interference under a sufficiently bright light source (Figure S1). The detailed outcomes are listed in Table 2.

### 2.5. Safety

We evaluated the safety of 7-MEGA^TM^ by collecting data on adverse effects and performing a relevance assessment of the consumption of 7-MEGA^TM^. We also conducted laboratory tests (complete blood count, blood chemistry, and urine) and collected the vital signs (blood pressure, pulse, and body temperature) of the participants.

### 2.6. Sample Size Calculation

To calculate the minimum number of participants required, we referred to the results of Hwang et al. [20] and used the R4 parameter of skin wrinkles measured by device analysis. Through this process, it was concluded that to achieve a significance level of 5% and power of 84%, the required sample size was 40 participants in each group. Considering the dropout rate of 20%, it was planned to register 50 participants per group (=40/[1 − 0.2]), a total of 100 participants.

The sample size calculation formula for hypothesis testing was as follows:

(1) $$n_{c}=\frac{2{\left(Z_{1-\alpha /2}+Z_{1-\beta }\right)}^{2}{\sigma_{1}}^{2}}{{\left(D_{1t}-D_{1c}\right)}^{2}}=\frac{2{\left(1.960+0.9945\right)}^{2}0.03^{2}}{{\left(-0.02\right)}^{2}}\;\approx 40,$$

where $D_{1t}$ is the change in R4 after consuming the test food, and $D_{1c}$ is the change in R4 after consumption of the control food.

### 2.7. Statistical Analyses

For continuous variables, the number of participants, mean, and standard deviation were used, and for categorical variables, frequencies and percentages were used. Comparisons between groups of continuous variables were performed using the unpaired *t*-test or Wilcoxon rank-sum test, depending on normality, and comparisons within groups were performed with the paired *t*-test or Wilcoxon signed-rank test, depending on normality. Comparisons of categorical variables between groups were performed using Pearson’s chi-square test or Fisher’s exact test, and within-group comparisons were performed using McNemar’s test. Demographic information, medication, and medical history data were obtained from the patients in the per-protocol set (PPS) analysis group.

Functional evaluation was conducted after completing the test, and the PPS group was analyzed. The PPS included participants who completed the trial with an intake compliance rate of more than 80% and had no major protocol violations affecting the human application test results. The full analysis set group included participants who consumed the product at least once, underwent functional evaluation at least once, and did not violate the main selection criteria, and was used as the secondary analysis group. Safety evaluation was conducted on the safety analysis set group who consumed at least one IP and simultaneously received a safety assessment at least once. All variables were evaluated in the left and right positions, and analyses were conducted for each position. Both cheeks and forearms were examined for skin and transepidermal moisture loss.

For the primary and secondary evaluation variables, the mean and standard deviation were analyzed for each group (*p* < 0.05, two-sided). The least squares mean (LSmean) and standard error for the change in measurements at 12 weeks (Visit 5) compared to the baseline (Visit 2) measurements, corrected through a statistical model, were analyzed for each group. Differences between the groups in the change in measurements at 12 weeks (Visit 5) compared to baseline (Visit 2), mean, standard deviation, and two-sided 95% confidence intervals were calculated, and the least squares average between the two groups was calculated using a statistical model. Differences (LSmean differences) and two-sided 95% confidence intervals were analyzed. Comparisons between groups were performed by fitting an analysis of covariance (ANCOVA) with the change in measurements at 12 weeks (Visit 5) compared to baseline (Visit 2) as a response variable and the baseline (Visit 2) measurements as a covariate. If additional factors affecting the outcome variables were identified, the general linear model, which is an extension of the ANCOVA model, was fitted by adding relevant factors as covariates. When the parametric method was not appropriate owing to data bias, the Wilcoxon rank-sum test was used for analysis.

## 3. Results

### 3.1. Participant Characteristics

The first participant was screened on 6 March 2023, and the last completed the study on 19 June 2023. A screening evaluation was conducted on 109 females to select the appropriate participants. Of these, eight participants were excluded, and 101 participants (50 in the 7-MEGA^TM^ group and 51 in the control group) were randomly assigned. Of these, three dropped out of the 7-MEGA^TM^ group, and a total of 98 participants completed the human application test (47 in the 7-MEGA^TM^ group and 51 in the control group).

During participation, three participants were excluded, including one participant for whom the functional evaluation was not conducted due to withdrawal of consent, one participant who was a smoker, and one whose contact information was lost and tracking failed. These participants were all from the intervention group. Following the intervention, two participants were additionally excluded, including one participant in the intervention group who used a prohibited drug in combination with a steroid, and one participant in the control group whose overall intake compliance was less than 80%. Finally, 96 participants (46 in the 7-MEGA^TM^ group and 50 in the control group) were included in the analysis.

The baseline demographic information of the participants was similar in terms of age (7-MEGA^TM^ group: 45.63 ± 4.11 years; control group: 47.12 ± 4.41 years). Participants in the 7-MEGA^TM^ group were significantly taller than participants in the control group (161.31 ± 5.80 cm vs. 158.39 ± 4.25 cm, *p* = 0.0065). This height difference was not taken into account when analyzing the functional evaluation variables, as it was deemed not to affect the evaluation.

Additionally, there were no significant differences between the two consumption groups regarding weight, skin treatment, and skin care. The detailed information is listed in Table 3.

### 3.2. Study Endpoints

**Figure 1:**
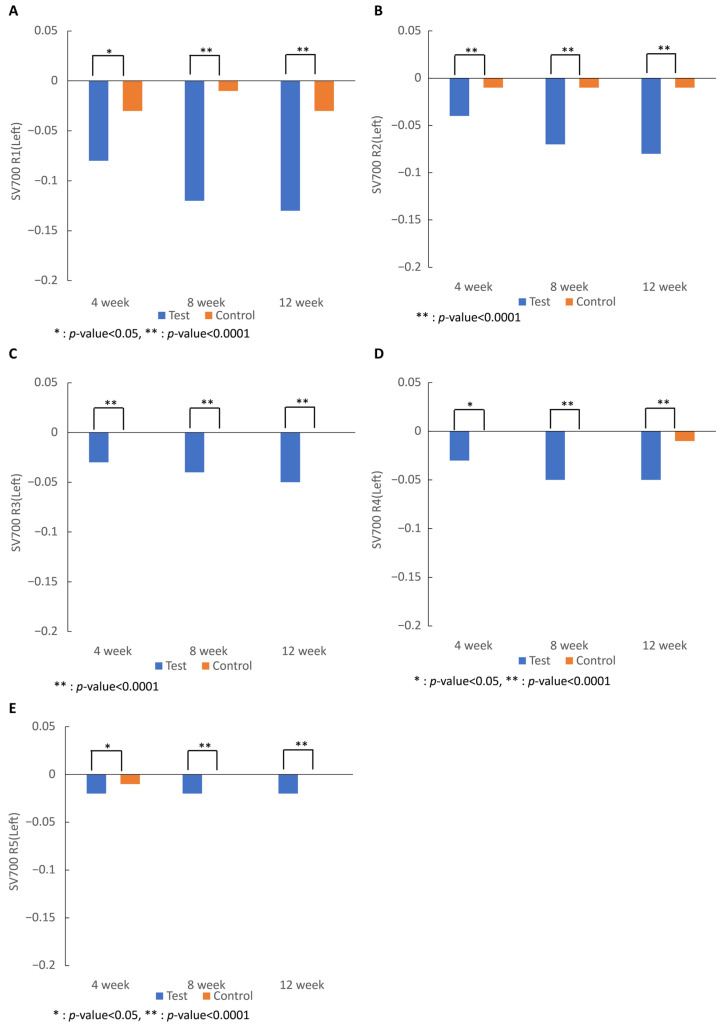
Effect of 7-MEGA^TM^ on skin wrinkle parameters: (**A**) skin roughness; (**B**) maximum roughness; (**C**) average roughness; (**D**) smoothness depth; (**E**) arithmetic roughness average measured by the SV700 at 4 weeks, 8 weeks, and 12 weeks. * indicates that the *p*-value between the two groups is <0.005. ** indicates that the *p*-value between the two groups is <0.0001.

Skin wrinkles measured in R1 to R5 values on both sides of the right eye area in R1, R2, R3, R4, and R5 in the test group began to decrease significantly after 4 weeks of intake (R1, R4, and R5, *p* < 0.05; R2 and R3, *p* < 0.0001) and significantly decreased compared to those in the control group after 12 weeks of intake (R1–5, *p* < 0.0001), showing that skin wrinkles were significantly improved by the intake of 7-MEGA^TM^ (Figure 1).

As a result of analyzing the moisture content on both sides, the test group began to show a significant increase after 4 weeks of intake (*p* < 0.0001), and the moisture content after 12 weeks of intake compared to before intake was significantly higher than that of the control group (*p* < 0.0001), showing that skin moisture was significantly improved by the intake of 7-MEGA^TM^.

**Figure 2:**
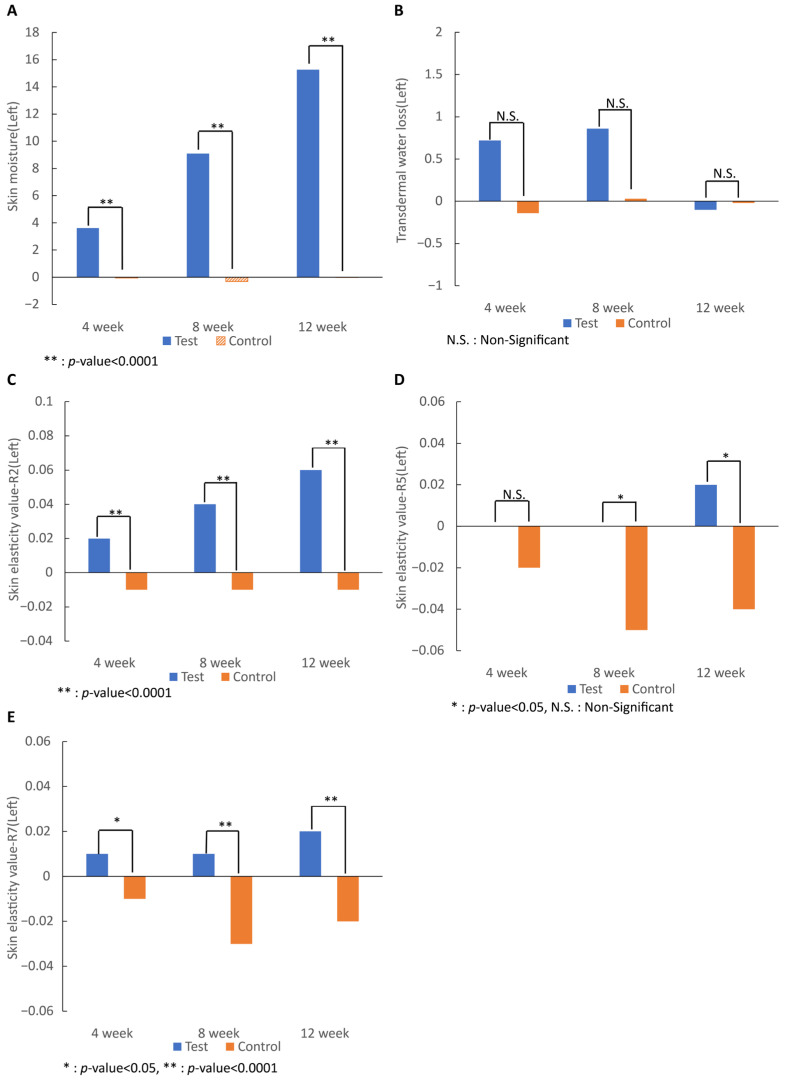
Effect of 7-MEGA^TM^ on (**A**) skin moisture and (**B**) transdermal water loss, and skin elasticity values of (**C**) total elasticity, (**D**) real elasticity, and (**E**) ratio of elasticity to the entire curve at 4 weeks, 8 weeks, and 12 weeks. * indicates that the *p*-value between the two groups is <0.005. ** indicates that the *p*-value between the two groups is <0.0001. N.S. indicates that the *p*-value between the two groups is statistically non-significant.

The transepidermal water loss value of both cheeks decreased after 12 weeks of intake compared with that before consumption, but the difference was not significant. Skin elasticity of the R2, R5, and R7 values on both sides revealed that the 7-MEGA^TM^ group showed a tendency of increased values in all categories from 4 weeks after intake (R2, *p* < 0.0001; R5, N.S; and R7, *p* < 0.05). After 12 weeks, the skin elasticity of the 7-MEGA^TM^ group was significantly higher than that of the control group (R2 and R7, *p* < 0.0001; R5, *p* < 0.05), except for R5 on the right side (Figure 2).

Melanin analysis confirmed that all values in the test group began to decrease after 4 weeks of intake, and the change in the erythema index after 12 weeks was significantly lower than that in the control group (*p* < 0.0001). Analysis of the erythema index showed that for all values, both groups had increased values after 4 weeks of intake, and the change in the erythema index after 12 weeks showed no significant differences after 7-MEGA^TM^ intake compared to that of the control food.

**Figure 3:**
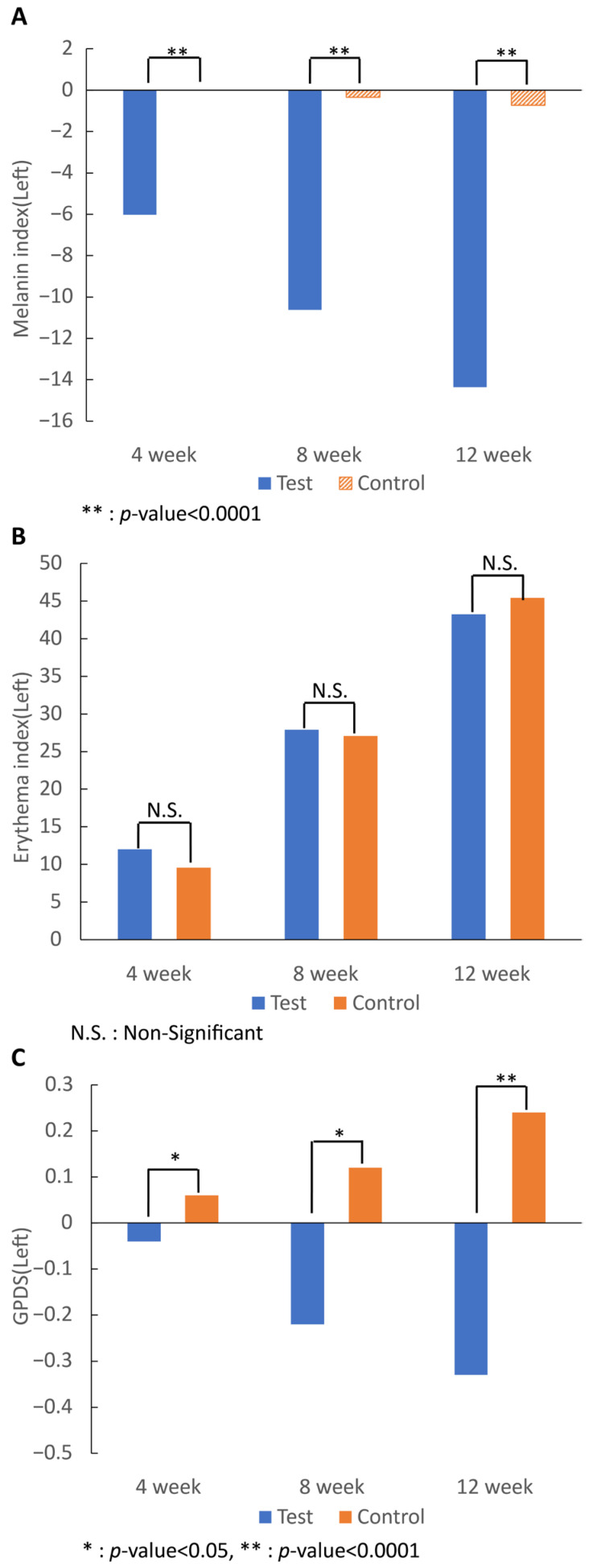
Effect of 7-MEGA^TM^ on: the (**A**) melanin; (**B**) erythema indexes; and (**C**) GPDS at 4 weeks, 8 weeks, and 12 weeks. * indicates that the *p*-value between the two groups is <0.005. ** indicates that the *p*-value between the two groups is <0.0001. N.S. indicates that the *p*-value between the two groups is statistically non-significant.

Regarding the change in the GPDS score on both sides, the score of the 7-MEGA^TM^ group began to decrease after 4 weeks of intake (*p* < 0.05), and the value after 12 weeks was reduced compared to that of the control group (*p* < 0.0001) (Figure 3).

### 3.3. Safety

In the intervention group, seven adverse events occurred in six (12.50%) participants, and in the control group, 12 adverse events occurred in 10 (19.61%) participants. There were no significant differences in the number of adverse events between the 7-MEGA^TM^ group and control groups (*p* = 0.3370, chi-square test). However, there were no adverse drug reactions or serious adverse reactions directly related to the test food.

The seven adverse events that occurred in the test group were pharyngitis (two cases), rhinitis, cystitis, allergic reactions, indigestion, and itching. Of the 12 adverse events that occurred in the control group, pharyngitis occurred most frequently (six cases in five patients [9.80%]), followed by sinusitis, pyelonephritis, back pain, muscle pain, menstrual pain, and headache (one case each). All adverse reactions were mild and unrelated to foods for human application testing. In terms of laboratory testing, most results did not change significantly compared to before intake, and even if there were changes, they were all within the normal range and had no significant clinical significance. There was no significant difference in terms of the degree of change between the two groups.

### 3.4. Physical Activity and Diet

We analyzed the lifestyle habits of the participants before the trial (Visit 1) and at the last visit (Visit 5), such as smoking status, alcohol consumption status, coffee consumption, outdoor activity time, makeup-application frequency, sleep time, and frequency of sunscreen use. There were no meaningful differences in any of the lifestyles observed between the two groups between Visits 1 and 5.

We conducted an analysis of a 24 h recall dietary survey of the participants and calculated the total calories before intake (Visit 2) and at 4 weeks (Visit 3), 8 weeks (Visit 4), and the end of intake (Visit 5). There were no significant differences between groups at any time point. The lifestyle habits of participants are summarized in Table 4.

## 4. Discussion

The test food used in this trial, 7-MEGA^TM^, is a raw material containing PA. PA is an unsaturated fatty acid with a double bond at the seventh carbon and is found in the lipid bilayer of all human tissue cell membranes that participate in multiple metabolic processes [21]. PA is found in oils extracted from animals, plants, and aquatic products [22]. Specifically, PA can also be obtained from products such as macadamia nuts, mink oil, and dairy [23], but in this study, PA extracted from defined Alaska pollock fish oil, called 7-MEGA^TM^, was used as a test food.

The anti-inflammatory and antioxidant action of 7-MEGA^TM^ on the skin has been proven in previous studies. An in-vitro study showed that 7-MEGA^TM^ has an anti-inflammatory effect on high sensitivity of human epidermal keratinocyte cells, which promote collagen regeneration in the presence of cytotoxicity caused by hydrogen peroxide, and the anti-inflammatory effect of 7-MEGA^TM^ appears to be mediated through silent information regulator 1 activation [24]. In addition, a clinical study conducted in humans reported that 7-MEGA^TM^ not only restored skin barrier function but also improved quality of life by safely improving skin elasticity and wrinkles [25]. Compared to previous studies, we adopted more outcomes, such as wrinkles, melanin index, erythema index, and GPDS, to clarify the efficacy of 7-MEGA^TM^ on skin aging.

Fatty acids are oxidized by the mitochondria into acetyl-coenzyme A units [26]. Well-known unsaturated fatty acids, such as omega-3, 6, 9, and 12, are vulnerable to oxidation as polyunsaturated fatty acids have several double bonds; however, PA, a monounsaturated fatty acid, is relatively resistant to the oxidation process [27]. In addition, 7-MEGA^TM^ has its own stability increased by the addition of antioxidants during manufacturing. Distilling, transesterification, and chilling are performed to remove impurities and saturated fatty acids from the extracted fish oil, and then natural mixed tocopherols are added before packaging. The final product passed the long-term stability test for 3 years [28].

This study had some limitations, the first of which is that it was conducted at only one center, making it difficult to generalize the data to different populations. Additionally, because it targeted only middle-aged females, the safety and effectiveness of long-term use in young females without wrinkles could not be determined. In the future, follow-up multicenter and cross-sectional studies on the skin improvement effect of 7-MEGA^TM^ will need to be conducted. In addition, there is sufficient evidence for the positive effect of the majority of unsaturated fatty acids on the body regarding test raw materials, but few studies have yet fully compared the efficacy of omega-7 and omega-3 or -6.

Skin wrinkles were measured using the SV700 in the R1 to R5 values, and all values in the 7-MEGA^TM^ group significantly decreased compared to those in the control group after 4 and 12 weeks of intake. The GPDS score, a subjective evaluation of wrinkles, of the 7-MEGA^TM^ group decreased significantly compared to that of the control group, showing similar trends to the results of skin wrinkle evaluation using the SV700. It can be concluded that wrinkles of the 7-MEGA^TM^ group improved in both objective evaluation through the SV700 and subjective evaluation through expert judgment.

Skin moisture measured with the corneometer showed significant increases in all values 4 and 12 weeks after intake, consistent with a previous study by Koh et al. [25], which means that oral administration of 7-MEGA^TM^ contributed to the prevention of skin aging through hydration in both studies.

Transepidermal water loss was measured using the tewameter. Not only was there no significant result at all, but at weeks 4 and 8, the test group showed more transdermal water loss than the control group. Although taking the test food significantly increased the moisture content of the skin itself, oral administration of 7-MEGA^TM^ did not seem to have prevented the increased loss of moisture. In a previous study using a two-meter TM-300, as in this study, the amount of transdermal water loss in the test group decreased, but it was not significant [25].

Analysis of skin elasticity measured by cutometer in the R2, R5, and R7 values showed that the 7-MEGA^TM^ group had improved values compared with that of the control group after 12 weeks of intake.

The melanin and erythema index were measured through the same mexameter, but the opposite result was shown. The melanin index in the test group was significantly decreased at 4, 8, and 12 weeks; however, the erythema index of the test group showed no significant differences after 12 weeks. Long-term use of PA prevented melanin pigmentation in the skin but did not seem to increase blood flow in the skin.

In this clinical trial, no significant difference in the incidence of adverse events was observed between the two groups after product consumption, and there were no clinically significant changes in laboratory tests. Therefore, 7-MEGA^TM^ was considered safe, even for long-term consumption.

## 5. Conclusions

We conducted a randomized clinical trial for the oral consumption of 7-MEGA^TM^, a food manufactured from refined Alaskan pollock fish oil containing omega-7. In the group that consumed 7-MEGA^TM^, anti-aging effects on skin, including improvement in wrinkles and skin moisture, loss of elasticity, and reduced melanin and erythema production were observed.

No adverse drug reactions or serious adverse reactions occurred during the study period related to the product. In laboratory tests, changes before and after consumption of the test food were within the normal range, with minimal detectable changes and no significant changes.

In conclusion, 7-MEGA^TM^ was shown to improve skin health through the skin wrinkle, skin moisturizing, and skin elasticity function improvement effects and the melanin index improvement effect, and had positive safety results, as confirmed through human application tests. It may be considered a safe raw material and may be useful as a skin nutritional supplement.

## Figures and Tables

**Table 1:** Eligibility and exclusion criteria.

Inclusion Criteria
(1) Females between 40 and 59 years of age (2) Females with a Global Photo Damage Score around the eyes over 3 (3) Females whose water retention in both cheeks measured with a corneometer (Corneometer CM 825; Courage + Khazaka Electronic GmbH, Köln, Germany) at Visit 1 and Visit 2 is less than 49 AU (4) Females who are not currently taking functional foods or products to improve skin health, such as for wrinkles, elasticity, moisturizing, or to reduce melanin and erythema production (5) Females who are able to provide written informed consent
**Exclusion Criteria**
(1) Females with severe heart, immune, gastro-intestinal, liver, kidney, nervous system, musculoskeletal, or infectious diseases or malignant tumors who currently receive treatment (2) Females with a psychological history or currently suffering from schizophrenia, depression, drug addiction, alcohol use/induced disorders, etc. (3) Females with skin diseases, such as atopic dermatitis, and psoriasis or skin abnormalities, such as spots, acne, erythema, or capillary dilatation at the measurement area (4) Smokers or participants who quit smoking less than 1 year prior to the study (5) Females who underwent skin wrinkle removal procedures, such as Botox or fillers, within 6 months, or skin peeling within 1 month before the study (6) Females who consumed oral or applied retinoids, steroids, etc., within 3 months before the study (7) Females who took drugs for inhibiting obesity (fat absorption inhibitors, appetite suppressants, etc.), psychiatric drugs (depression or schizophrenia), or diuretics within 1 month before the study (8) Females who consumed functional foods or medicine aimed at improving skin wrinkles and moisture (including antioxidants; hyaluronic acid; collagen; evening primrose oil; vitamins A, C, or E) within 2 weeks before the study (9) Females who used functional cosmetics to improve skin wrinkles (retinoids, alpha hydroxy acids, etc.), highly moisturizing cosmetics, or skin care devices (light emitting diode masks, ion boosters, etc.) within 2 weeks before the study (10) Females with uncontrolled hypertension (systolic blood pressure >160 mmHg or diastolic blood pressure > 100 mmHg, measured after 10 min of rest) or uncontrolled diabetes (fasting blood sugar > 180 mg/dL) (11) Females whose aspartate aminotransferase and alanine transaminase levels are more than 3 times the normal range and serum creatinine levels are more than 2 times the normal range, which prevents them from participating in the study, according to the opinion of an expert (12) Females who had participated in other clinical trials for skin health within 3 months prior to the study (13) Females who are pregnant, lactating, or planning pregnancy and those using contraceptives or female hormones (14) Females with a history of hypersensitivity (allergy) to omega-7 (15) Other females who are deemed unsuitable for the study according to the judgment of the principal investigator

**Table 2:** Primary and secondary evaluation parameters.

Primary Outcomes
(1) Change in skin wrinkles (R1: skin roughness, R2: maximum roughness, R3: average roughness, R4: smoothness depth, and R5: arithmetic roughness average) measured with the SV700 (Courage + Khazaka Electronic GmbH) at 12 weeks compared to baseline (2) Change in skin moisture measured with the corneometer at 12 weeks compared to baseline
**Secondary Outcomes**
(1) Change in skin wrinkles (R1, R2, R3, R4, and R5) measured with the SV700 at 4 and 8 weeks compared to baseline (2) Change in skin moisture measured with the corneometer at 4 and 8 weeks compared to baseline (3) Change in transepidermal water loss measured with the tewameter at 4, 8, and 12 weeks from baseline (4) Change in skin elasticity (R2: total elasticity, R5: real elasticity, and R7: the ratio of elasticity to the entire curve) measured with the cutometer at 4, 8, and 12 weeks compared to baseline (5) Changes in the melanin and erythema indexes measured with the mexameter at 4, 8, and 12 weeks from baseline (6) Changes in GPDS at 4, 8, and 12 weeks compared to baseline

**Table 3:** Demographic information of the participants.

		Intervention (*N* = 46)	Control (*N* = 50)	*p*-Value
Sex, *N* (%)				-
	Female	46 (100.0)	46 (100.0)	
	Male	0 (0.00)	0 (0.00)	
Ethnic group, *N* (%)				-
	Asian	46 (100.0)	46 (100.0)	
	Non-Asian	0 (0.00)	0 (0.00)	
Age (year)				0.0713
	Mean ± SD	45.63 ± 4.11	47.12 ± 4.41	
	Min, Max	40.00, 53.00	40.00, 54.00	
Height (cm)				0.0065
	Mean ± SD	161.31 ± 5.80	158.39 ± 4.25	
	Min, Max	150.90, 174.00	150.10, 170.10	
Weight (kg)				0.1206
	Mean ± SD	61.11 ± 8.66	58.56 ± 7.28	
	Min, Max	43.10, 83.10	44.90, 74.40	
Skin treatment or skin care, *N* (%)				-
	Ongoing	0 (0.00)	0 (0.00)	
	None	46 (100.0)	46 (100.0)	

**Table 4:** Comparison of lifestyle habits of participants in baseline.

		Intervention (*N* = 46)	Control (*N* = 50)	*p*-Value
Smoking status, *N* (%)				-
	Non-smoker	46 (100.0)	50 (100.0)	
	Ex-smoker	0 (0.00)	0 (0.00)	
	Current smoker	0 (0.00)	0 (0.00)	
Alcohol consumption, *N* (%)				0.8033
	Drinker	33 (71.74)	37 (74.00)	
	Non-drinker	13 (28.26)	13 (26.00)	
Coffee consumption, *N* (%)				1.0000
	Drinker	42 (91.30)	46 (92.00)	
	Non-drinker	4 (8.70)	4 (8.00)	
Outdoor activity time/day, *N* (%)				0.1755
	<3 h	38 (82.61)	34 (68.00)	
	3–5 h	4 (8.70)	11 (22.00)	
	>5 h	4 (8.70)	5 (10.00)	
Makeup-application frequency, *N* (%)				0.2414
	0	15 (32.61)	20 (40.00)	
	1–2	10 (21.74)	15 (30.00)	
	3–4	13 (28.26)	6 (12.00)	
	5–7	8 (17.39)	9 (18.00)	
Average sleep time per day, *N* (%)				0.6575
	<5 h	0 (0.00)	2 (4.00)	
	5–8 h	41 (89.13)	43 (86.00)	
	>8 h	5 (10.87)	5 (10.00)	
Sunscreen use per week, *N* (%)				0.4404
	0	5 (10.87)	2 (4.00)	
	1–2	5 (10.87)	9 (18.00)	
	3–4	6 (13.04)	9 (18.00)	
	5–7	30 (65.22)	30 (60.00)	

## Data Availability

The data are not publicly available due to large amount of original data.

## Supplementary Materials

The following supporting information can be downloaded at: https://www.mdpi.com/article/10.3390/nu16020212/s1, Figure S1: Rating criteria of GPDS.
