# Supplementary figures and images for: Anti-Wrinkle and Skin Moisture Efficacy of 7-MEGATM: A Randomized, Double-Blind, Placebo Comparative Clinical Trial
Source: Nutrients. 2024 Jan 9;16(2):212. doi: 10.3390/nu16020212 (PMC10819616; doi:10.3390/nu16020212)

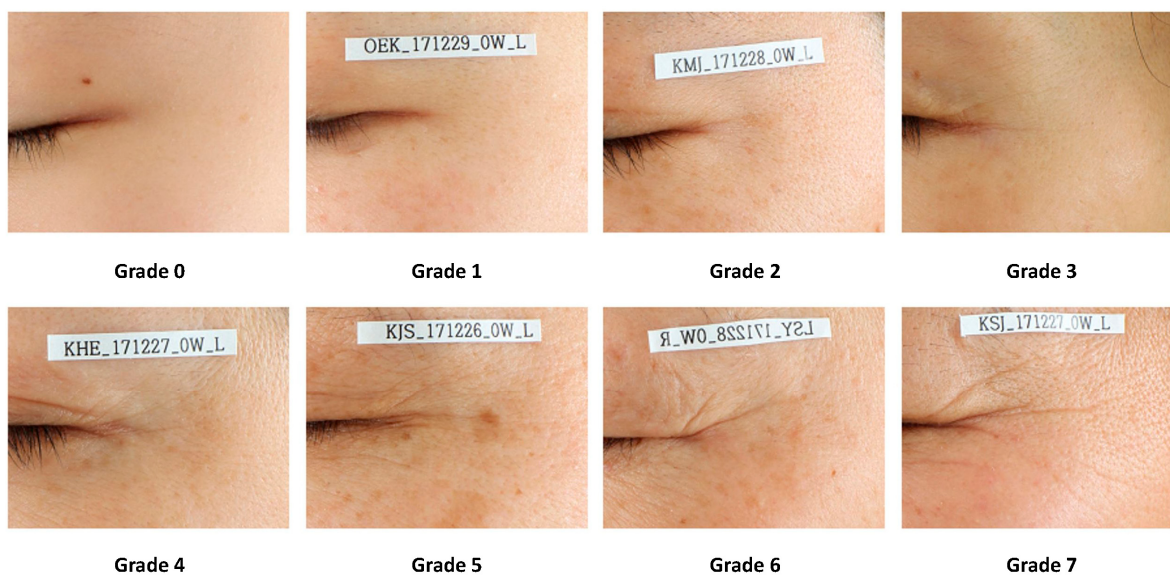

Figure S1: Rating criteria of GPDS.

Supplement: Supplementary file 1 [file nutrients-16-00212-s001.zip › nutrients-2758822-supplementary.pdf]
